# Well-being during COVID-19 pandemic in Russia: The effects of defensive optimism, destructive coping and gender

**DOI:** 10.1192/j.eurpsy.2021.1607

**Published:** 2021-08-13

**Authors:** T. Gordeeva, O. Sychev, M. Lunkina

**Affiliations:** 1 Psychology, Lomonosov Moscow State University, Moscow, Russian Federation; 2 Research Department, Shukshin Altai State University for Humanities and Pedagogy, Moscow, Russian Federation

**Keywords:** destructive coping, well-being, COVID-19 pandemic, Defensive optimism

## Abstract

**Introduction:**

Previous research shows that subjective well-being during pandemic (SWB-P) is related to sociodemographic variables (de Pedraza et al., 2020) and coping (Rasskazova et al., 2020). We hypothesized that SWB-P depends on specific types of optimism and coping with pandemic situation, namely defensive optimism (belief the coronavirus problem is exaggerated) and constructive optimism (belief that people’s efforts help prevent infection and spread of the virus) which effects are mediated by the effects of destructive and constructive coping.

**Objectives:**

This study aimed to assess the effects of situation specific optimism and coping on SWB controlling for gender.

**Methods:**

The sample comprised 1403 university students (68% women, M=20.59, SD=3.66). Online survey has been conducted from 10/4/2020 till 25/4/2020. The measures included LOT-R, the scales of defensive and constructive optimism, and the scales of destructive and adaptive coping with pandemic situation (Gordeeva, Sychev, 2020). Well-being was assessed by sum of positive affect minus negative affect (PANAS) and SWLS (Diener et al., 1985).

**Results:**

Structural equation modeling shows that SWB-P is related directly to gender (less well-being in women), dispositional optimism, adaptive coping and destructive coping (negatively). The negative effect of defensive optimism was mediated only by destructive coping (p<0.001), the effects of constructive optimism on well-being was mediated by adaptive and destructive coping (both p<0.01) (χ2 (4)= 8.97; p = 0.06; CFI = 0.996; TLI = 0.978; RMSEA = 0.030; PCLOSE = 0.886).

**Conclusions:**

Dispositional optimism together with situation-specific defensive and constructive types of optimism and coping are essential for explaining well-being during Covid-19 lockdown.

